# The Role of Body Image Discrepancy in Exercise and Eating Motivation: A Self-Determination Theory Perspective

**DOI:** 10.3390/healthcare14111445

**Published:** 2026-05-23

**Authors:** Rogério Salvador, Filipa Cordeiro, Ruth Jimenéz Castuera, Ricardo Rebelo-Gonçalves, Diogo Monteiro

**Affiliations:** 1 ESECS, Polytechnic University of Leiria, 2411-901 Leiria, Portugal; fi_cordeiro@hotmail.com (F.C.); ricardo.r.goncalves@ipleiria.pt (R.R.-G.); diogo.monteiro@ipleiria.pt (D.M.); 2 Research Center in Sport Sciences, Health Sciences and Human Development (CIDESD), 2411-901 Leiria, Portugal; 3 Life Quality Research Center (CIEQV), Polytechnic University of Leiria, 2411-901 Leiria, Portugal; 4 Faculty of Sport Sciences, University of Extremadura, 10003 Cáceres, Spain; ruthji@unex.es; 5 Research Unit for Sport and Physical Activity (CIDAF), University of Coimbra, 2040-248 Coimbra, Portugal

**Keywords:** body image discrepancy, motivation, exercise, dietary behavior, sex, self-determination theory, Stunkard figure rating scale

## Abstract

**Background/Objectives:** While body dissatisfaction is frequently studied as an outcome of physical activity, less is known about how pre-existing body image perceptions shape the quality of behavioral regulation. Grounded in Self-Determination Theory, this study aimed to investigate the associations of different perceived body image discrepancy profiles with autonomous and controlled motivation for both exercise and eating, and to explore the interaction effects between these profiles and sex. **Methods:** The sample comprised 939 regular gym exercisers (32.99 ± 11.90 years; 55.1% female). Using the Stunkard Figure Rating Scale, participants were categorized into four discrepancy profiles: desire to increase, satisfied, mild desire to reduce, and moderate/severe desire to reduce. Data were analyzed using Two-Way ANOVAs. Results: The Satisfied group reported the highest autonomous and lowest controlled motivation across both domains (main effects: *p* < 0.001, η^2^_p_ = 0.019–0.046). A significant body image × sex interaction emerged for controlled eating motivation (F(3, 931) = 6.22, *p* < 0.001, η^2^_p_ = 0.020). Females exhibited a “U-shaped” curve, demonstrating low controlled eating motivation when satisfied (M = 1.65) but elevated levels at extremes (desire to increase: M = 2.50; moderate/severe desire to reduce: M = 2.39). Males maintained stable controlled eating motivation across all discrepancy profiles (M = 2.06–2.30). Although these main and interaction effects were statistically significant, all observed multivariate effect sizes were small (η^2^_p_ = 0.012–0.046). **Conclusions:** Perceived body image discrepancy acts as a significant antecedent of motivational quality. The absence of a perceptual gap is linked to highly adaptive, autonomous behavioral regulation. Furthermore, the distinct sex-based patterns in controlled eating motivation underscore the necessity for health and exercise professionals to adopt tailored, sex-specific strategies when addressing body image concerns.

## 1. Introduction

Regular exercise and the maintenance of an active lifestyle are consistently associated with health benefits [1]. However, despite the widespread recognition of these positive outcomes, physical activity adherence remains low across diverse populations, with only a minority of individuals consistently meeting recommended guidelines over time [2,3,4]. Globally, insufficient physical activity represents a major public health concern. Current estimates indicate that one in four adults and three in four adolescents do not meet the physical activity guidelines established by the World Health Organization [5].

In Portugal, data indicate that 73% of the population is insufficiently active, with a lack of time (44%) and low motivation (29%) identified as primary barriers to engagement [6]. This high prevalence of physical inactivity is linked to public health challenges, including an increased prevalence of chronic diseases, reduced physical fitness, and compromised psychological well-being [4]. Consequently, promoting regular physical activity, either through structured exercise programs or the integration of active daily routines, is a relevant strategy to improve both physical and mental health outcomes at the population level [7,8].

### 1.1. Body Image

Motivation and behavioral persistence are influenced by multiple psychological factors, including how individuals perceive and evaluate their own body image [9]. Body image is conceptualized as a multidimensional construct that encompasses perceptual, cognitive, affective, and behavioral components [10]. Within this broad framework, a main area of investigation is the perceived discrepancy between an individual’s self-perceived current body figure and their internalized ideal body figure [11]. This perceptual–evaluative gap reflects a subjective cognitive judgment [11] and represents a relevant psychological factor associated with psychological well-being and health-related behaviors [12].

When individuals experience significant desynchronization between their actual and desired body size, they often report higher levels of psychological distress, including body-related shame, social appearance anxiety, and insecurity [13,14]. In health contexts, these negative affective experiences are linked to maladaptive behavioral patterns, such as rigid or dysfunctional eating and exercise practices, frequently driven by internal or external appearance-related pressures [15]. Although regular physical activity has the potential to improve body image evaluations over time [16], a pre-existing perceived body image discrepancy may fundamentally alter the psychological lens through which individuals regulate their behavioral motivation.

Understanding this perceptual gap is essential, as the direction and magnitude of the discrepancy can differentially shape motivational regulation across health domains [10]. While a substantial body of the literature has explored body dissatisfaction as an outcome, fewer studies investigate how a pre-existing discrepancy acts as an antecedent that relates to distinct motivational profiles in both exercise and eating behaviors, particularly when considering potential variations between sexes. By isolating this specific perceptual–evaluative component, this study aims to examine how the subjective gap between the current and ideal body interfaces with the quality of motivation, providing insights into the psychological mechanisms that underpin health behaviors in gym settings.

### 1.2. Self-Determination Theory

Self-Determination Theory (SDT) [17] is a comprehensive macro-theory of human behavior that provides a framework for understanding the quality of motivation in health contexts [18]. Unlike cognitive models that focus primarily on behavioral planning and intention [19], SDT posits that motivation is not a unitary concept but exists along a continuum [20]. Within this continuum, behaviors can be regulated autonomously or controlled by internal or external pressures [17]. Autonomous motivation encompasses behaviors driven by inherent enjoyment (i.e., intrinsic motivation), integration into one’s identity (i.e., integrated regulation), or personal valuation of the outcomes (i.e., identified regulation) [21]. Conversely, controlled motivation involves behaviors enacted to obtain external rewards, avoid punishments (i.e., external regulation), or alleviate internal pressures such as guilt, shame, and ego-involvement (i.e., introjected regulation) and amotivation (i.e., a complete lack of intent) [22].

Research demonstrates that autonomous motivation is linked to better psychological health, positive affective responses, and long-term adherence [23,24]. In contrast, controlled motivation and amotivation represent the “dark side” of behavioral regulation, frequently associated with maladaptive outcomes, less enjoyment, and higher dropout rates [25]. The quality of an individual’s motivation is heavily influenced by their psychological and evaluative states, making SDT a suitable lens for exploring the behavioral impact of body image [26]. When individuals experience a perceived body image discrepancy, the resulting social physique anxiety and appearance-based comparisons [27] can act as powerful internal controllers. Furthermore, the fitness environment is inherently social and can often be perceived as evaluative or demanding [28,29,30]. For individuals already vulnerable due to a gap between their current and ideal bodies, these social and internal pressures can shift the regulatory focus away from the inherent value of the activity and toward appearance-driven, introjected motives [18,31].

This regulatory shift has implications for both exercise and dietary behaviors, which are interconnected within a broader lifestyle context [32]. In the exercise domain, acting out of body dissatisfaction typically fosters controlled motivation, increasing the risk of dysfunctional exercise patterns [21]. Similarly, in the nutritional domain, SDT highlights that eating behaviors can be autonomously regulated (e.g., eating for health, vitality, and genuine well-being) or controlled (e.g., rigid dieting driven by guilt or severe appearance concerns) [33]. Controlled eating regulation is strongly associated with maladaptive dietary patterns and psychological distress [34]. Therefore, an individual’s perceptual lens regarding their body image does not merely affect how they feel but relates to why they engage in both exercise and eating behaviors [26].

### 1.3. Present Study

Despite the robust framework provided by SDT, a critical gap remains in understanding how a pre-existing perceptual–evaluative gap, specifically, body image discrepancy, acts as an antecedent to motivational regulation across different health domains [18]. While previous research has established that body image is linked to motivational quality [26], most studies focus on body dissatisfaction as an outcome of exercise [16] or examine exercise and dietary behaviors in isolation. However, SDT suggests that motivational regulations can spill over across related life domains [32]. Furthermore, while sex differences in body image concerns and general motivation are acknowledged [35,36], there is limited understanding of how sex interacts with perceived body image discrepancy to differentially shape the quality of motivation for both exercise and eating. Addressing this gap is essential for developing more tailored and effective health promotion strategies. Therefore, the present study aims to investigate how different profiles of perceived body image discrepancy (i.e., ranging from the desire to increase body size and satisfaction to varying degrees of desire to reduce body size) are associated with autonomous and controlled motivation for both exercise and eating. Furthermore, this study aims to explore the interaction between these body image profiles and sex. Based on the theoretical framework, the following hypotheses were formulated:

**H1.**  *Regarding autonomous motivation for exercise, it is hypothesized that the satisfied group will report higher levels compared to the dissatisfied groups (i.e., desire to increase or reduce), with potential variations in this pattern depending on sex [21].*

**H2.**  *For controlled motivation for exercise, groups with pronounced dissatisfaction (i.e., desire to increase or moderate/severe desire to reduce) will report higher levels compared to the satisfied group, and the interaction with sex will be explored [22,31].*

**H3.**  *Regarding autonomous motivation for eating, the satisfied group is expected to present the highest levels. A main effect of sex is also anticipated, as well as potential interaction effects between sex and body image profiles [33,35].*

**H4.**  *For controlled motivation for eating, it is hypothesized that dissatisfaction groups will report higher levels. A significant interaction effect between body image groups and sex is expected, reflecting the distinct sociocultural pressures applied to males and females regarding body size and dietary behaviors [34,36].*

**Figure 1:**
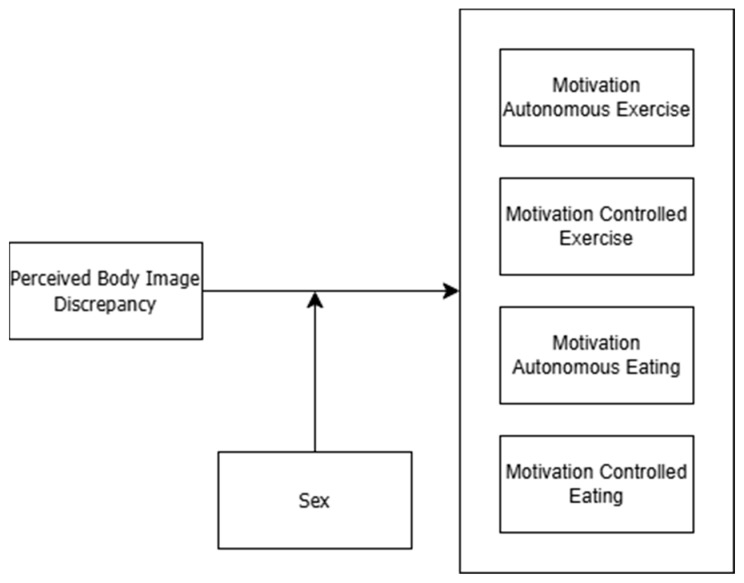
Conceptual model illustrating perceived body image discrepancy as an antecedent to exercise and eating motivation, with sex as a proposed moderator.

The conceptual model guiding these hypotheses is visually summarized in Figure 1, illustrating the positioning of the perceptual–evaluative gap as the primary antecedent, with sex acting as a potential moderator on the motivational outcomes.

## 2. Materials and Methods

### 2.1. Procedures

The study protocol was reviewed and approved by the Ethics Committee of the University of Extremadura (n.º 61/2022) prior to any data collection. Data collection was conducted in several fitness centers located in the central region of Portugal. This process was conducted under a formal cross-border academic and research collaborative partnership established between the Portuguese researchers’ institutions and the Faculty of Sport Sciences at the University of Extremadura (Spain), which oversaw and provided the ethical clearance for the study protocol. The recruitment process began with researchers contacting the management of several fitness centers. During these initial meetings, the study’s objectives, practical implications, and required procedures were explained, specifically requesting authorization to approach potential participants on-site, provided they met the inclusion criteria. Upon receiving formal approval from the club managers, researchers attended the facilities across various days and schedules. Potential participants were systematically approached at the gym entrance strictly before engaging in any fitness activity. This specific timing protocol was enforced to ensure that the acute physiological and psychological effects of a workout did not influence or bias the participants’ responses regarding their body image or motivation. To ensure privacy and concentration, the data collection took place in a closed, quiet environment (e.g., such as an initial physical assessment room) made available by the gym management. To maintain methodological consistency, all data were collected exclusively in person. Participants were given the option to complete the survey using either a traditional paper-and-pencil format or a digital format via Google Forms on a portable device provided by the researchers. Individuals who volunteered for the study first signed a written informed consent form. Subsequently, they completed the survey, which was structured in two parts: an initial section gathering sociodemographic information, followed by the specific questionnaires assessing the study variables described below. No compensation was provided for participation.

### 2.2. Participants

To ensure adequate statistical power, a power analysis was conducted a priori using G*Power 3.1.9.2 [37]. For a Multivariate Analysis of Variance (MANOVA), the input parameters included a small, expected effect size (f^2^(V) = 0.02), an alpha level of 0.05, and a statistical power of 0.95, considering the study’s factorial design and the dependent variables. This calculation yielded a minimum required sample size of 580 participants.

The inclusion criteria were: (i) having an active gym membership, excluding new members or individuals restarting after a significant break; and (ii) a minimum of six months of continuous structured exercise practice. The six-month threshold was established based on the Transtheoretical Model, as the maintenance phase is characterized by greater behavioral consistency and a significantly lower risk of dropout [8]. No restrictions were applied regarding the type of exercise modality (e.g., resistance training, group classes, or personal training) or training frequency (i.e., ranging from one to seven sessions per week).

### 2.3. Instruments

At the beginning of the survey, a sociodemographic questionnaire was administered solely for sample characterization purposes. This section collected self-reported data regarding the participants’ region, geographical zone, age, sex, height, weight, educational level, duration of exercise practice (in months or years), types of fitness activities practiced, and weekly training frequency.

To assess the several types of motivation for exercise, the Portuguese version of the Behavioral Regulation in Exercise Questionnaire (BREQ-3) [38] was used. This 24-item instrument builds upon Markland and Tobin’s earlier version [39] by incorporating the integrated regulation subscale developed by Wilson et al. [40]. Responses are recorded on a 5-point Likert scale ranging from 0 (completely disagree) to 4 (completely agree). The BREQ-3 captures the six motivational regulations proposed by SDT [17]. In accordance with the established SDT literature and methodological guidelines [20,31], these six regulations were aggregated into two composite macro-dimensions: autonomous motivation, comprising intrinsic, integrated, and identified regulations and controlled motivation, comprising introjected and external regulations, as well as amotivation. Previous studies robustly support the validity and reliability of this composite approach for assessing the quality of motivation [31,38]. In the current study, both composite dimensions demonstrated strong internal consistency (autonomous motivation α = 0.89; controlled motivation α = 0.91).

To evaluate the motivation regarding eating behaviors, the Portuguese version of the Regulation of Eating Behavior Scale (REBSp) [41] was administered. This 24-item questionnaire assesses eating motivation using a 7-point Likert scale ranging from 1 (strongly disagree) to 7 (strongly agree). Consistent with the exercise domain and the SDT theoretical framework [17,33], the items were combined into the same two macro-dimensions: autonomous motivation (i.e., intrinsic, integrated, and identified regulations) and controlled motivation (i.e., introjected, and external regulations, alongside amotivation). The REBSp has demonstrated solid psychometric properties in previous research in health and fitness contexts [41]. In the present sample, internal consistency was good for both factors (autonomous motivation α = 0.83; controlled motivation α = 0.86).

Perceived body image was assessed using the Stunkard Figure Rating Scale (FRS) [42], adapted for the Portuguese population [43]. This validated figural scale is widely used to measure perceptual and attitudinal aspects of body image [10,44]. Participants viewed a series of nine silhouettes (numbered from 1 to 9, progressing from the thinnest to the heaviest figure) and selected the silhouette that best represented their current body and, separately, the silhouette representing their ideal body. The core measure of interest was the perceived body image discrepancy score, calculated by subtracting the ideal figure rating from the current figure rating (Current—Ideal) [42].

To accurately capture both the direction (i.e., the desire to increase versus reduce body size) and the magnitude (i.e., mild versus moderate to severe) of body dissatisfaction, the discrepancy scores were initially operationalized into five distinct categories. This methodological approach avoids the loss of variance and the oversimplification associated with dichotomization, allowing for a comprehensive mapping of the perceptual–evaluative gap [44,45,46]. The five severity levels were defined as follows: (1) moderate to severe desire to increase body size (scores of −2 or lower); (2) mild desire to increase body size (score of −1); (3) satisfied with body size (score of 0, indicating no discrepancy); (4) mild desire to reduce body size (score of 1); and (5) moderate to severe desire to reduce body size (scores of 2 or higher). However, for the primary inferential analyses, and to ensure robust statistical power by avoiding highly unbalanced or small cell sizes, the two subgroups reflecting a desire to increase body size were merged. Consequently, participants were categorized into four final analytical groups: Desire to Increase (scores of −1 or lower); Satisfied (score of 0); Mild Desire to Reduce (score of 1); and Moderate/Severe Desire to Reduce (scores of 2 or higher). This refined operationalization directly addresses previous methodological criticisms by providing a nuanced comparison of how distinct body image profiles, across the full spectrum of dissatisfaction, interface with motivational regulation [11,47].

### 2.4. Data Analysis

All statistical analyses were performed using IBM SPSS Statistics for Windows, version 31.0 (IBM Corp., Armonk, NY, USA). Prior to the main analyses, data screening was conducted to address missing values and outliers. Questionnaires with missing data exceeding 5% were excluded from the dataset, and extreme multivariate outliers were identified and removed to ensure data quality. Given that all variables were self-reported and collected cross-sectionally, Harman’s single-factor test was conducted to diagnose potential Common Method Bias (CMB).

Assumptions for multivariate and univariate analyses were subsequently verified. Data normality was evaluated using the Kolmogorov–Smirnov test (n > 50). Homogeneity of covariance matrices was assessed using Box’s M test, while univariate homogeneity of variances was assessed using Levene’s test. In instances where Levene’s test indicated unequal variances, the analyses proceeded by relying on the robustness of ANOVA for large sample sizes, and appropriate robust post hoc adjustments were applied. In accordance with the Bonferroni correction, all omnibus *p*-values were evaluated against the adjusted threshold of 0.0125. All statistically significant main and interaction effects reported below comfortably survived these strict criteria (all *p* < 0.001). Additionally, a sensitivity power analysis was conducted retrospectively to determine the smallest detectable effect size given the final unbalanced group sizes achieved.

To examine the main effects of body image discrepancy profile, sex, and their interaction on the motivational outcomes, a two-way Multivariate Analysis of Variance (MANOVA) was initially performed. Following a significant omnibus MANOVA, follow-up two-way univariate ANOVAs were conducted for each dependent variable (i.e., autonomous, and controlled motivation for exercise and eating). To control the inflation of the family-wise error rate across the four univariate models, a Bonferroni correction was applied, establishing a conservative adjusted alpha threshold of 0.0125 (0.05/4) for determining statistical significance. significant main effects involving the four body image groups, post hoc pairwise comparisons were conducted utilizing Tukey’s HSD test or the Games-Howell test when the assumption of homogeneity of variances was not met. Effect sizes were calculated using partial eta-squared (η^2^_p_), with reference values of 0.01, 0.06, and 0.14 indicating small, medium, and large effects, respectively [48]. Finally, to provide a clearer estimate of the precision of the findings, 95% Confidence Intervals (CIs) were calculated and reported for key mean differences.

Finally, to ensure the robustness of the findings and to account for potential confounding variables, hierarchical multiple regression models were conducted. These models tested the predictive value of the continuous body image discrepancy score on the motivational outcomes, while explicitly adjusting for age, sex, and BMI in the first block. The standardized beta coefficients and 95% Confidence Intervals (CIs) from these adjusted models are provided in Appendix A Table A1.

## 3. Results

Prior to the main analyses, data screening procedures were conducted on the initial sample of 957 participants. The missing value analysis confirmed a complete dataset, with no participant exhibiting more than 5% missing data across the core measures. Multivariate outliers were evaluated using the Mahalanobis distance based on the four motivational variables. Participants exceeding the critical χ^2^ value of 18.46 (df = 4, *p* < 0.001) were identified as extreme multivariate outliers and subsequently removed from the dataset (n = 18). This procedure yielded a final, robust sample of 939 participants. To address potential concerns regarding common method variance associated with self-reported, cross-sectional data, Harman’s single-factor test was performed [49]. The unrotated principal component analysis of all items from the BREQ-3 and REBSp questionnaires revealed that the single largest factor explained only 24.10% of the total variance. Because this value falls well below the recommended threshold of 50%, CMB does not pose a threat to the validity of the current findings.

The final sample consisted of 939 regular gym exercisers (55.1% female, 44.9% male) with a mean age of 32.99 years (SD = 11.90, range: 18–65 years). Regarding educational attainment, the majority of the participants held a bachelor’s degree (46.3%) or completed secondary education (34.2%). In terms of training habits, participants reported an average exercise experience of 30.72 months (SD = 45.75) and a mean weekly training frequency of 3.76 sessions (SD = 1.28). The most prevalent primary exercise modality was independent resistance training (53.4%), followed by group classes (30.5%) and personal training (12.1%). A detailed description of the participants’ sociodemographic and training characteristics is presented in Table 1.

Descriptive statistics for autonomous and controlled motivation across both health domains (i.e., exercise and eating) were calculated and are presented in Table 2, organized by body image discrepancy profiles and sex. Overall, participants reported higher mean scores for autonomous motivation compared to controlled motivation in both the exercise and eating domains. When observing the body image profiles, a clear trend emerged. The satisfied group reported the highest levels of autonomous motivation and the lowest levels of controlled motivation for both exercise and eating. Conversely, participants at the extremes of the discrepancy spectrum (i.e., desire to increase and moderate/severe desire to reduce) exhibited higher mean scores in controlled motivation and lower scores in autonomous motivation. Regarding sex, females generally reported higher autonomous motivation for eating than males, while males presented slightly higher mean scores for controlled motivation in both domains.

To evaluate the study hypotheses (H1 to H4), four separate Two-Way ANOVAs were conducted to examine the main effects of body image discrepancy profile and sex, as well as their interaction, on each motivational variable. The assumption of homogeneity of variances (Levene’s test) was met for autonomous motivation for exercise (*p* = 0.249), controlled motivation for exercise (*p* = 0.986), and autonomous motivation for eating (*p* = 0.267). For controlled motivation for eating, this assumption was violated (*p* < 0.001). However, given the large sample size (N = 939), the MANOVA is robust to this violation, as detailed in the data analysis strategy.

Regarding exercise motivation, the results partially supported H1 and H2. A significant main effect of the body image profile was found for both autonomous (F(3, 931) = 8.97, *p* < 0.001, η^2^_p_ = 0.028) and controlled motivation (F(3, 931) = 11.02, *p* < 0.001, η^2^_p_ = 0.034). However, neither the main effect of sex nor the interaction between sex and body image profile reached statistical significance for exercise motivation. Post hoc Tukey HSD comparisons revealed that the Satisfied group reported significantly higher autonomous motivation compared to the moderate/severe desire to reduce group (*p* < 0.001). Conversely, for controlled motivation, the Satisfied group reported significantly lower levels compared to both the desire to increase (*p* < 0.001) and moderate/severe desire to reduce (*p* < 0.001) groups. While statistically significant, these effect sizes are considered small. In the eating domain, H3 was supported. Significant main effects were observed for both body image profile (F(3, 931) = 5.89, *p* < 0.001, η^2^_p_ = 0.019) and sex (F(1, 931) = 11.74, *p* < 0.001, η^2^_p_ = 0.012) on autonomous motivation for eating. Females reported higher overall autonomous regulation than males, and the satisfied group exhibited significantly higher autonomous motivation than the moderate/severe desire to reduce group (*p* < 0.001). The interaction effect was not significant (*p* = 0.450). Addressing H4, a significant main effect of body image profile (F(3, 931) = 14.90, *p* < 0.001, η^2^_p_ = 0.046) was found for controlled eating motivation, alongside a highly significant interaction effect between body image profile and sex (F(3, 931) = 6.22, *p* < 0.001, η^2^_p_ = 0.020). The main effect of sex alone was not significant (*p* = 0.376). This interaction indicates that the impact of body image discrepancy on controlled eating behaviors operates differently for males and females. Descriptive analysis reveals that while males maintain relatively stable levels of controlled eating motivation regardless of their body image satisfaction, ranging from 2.06 to 2.30, females exhibit a “U-shaped” curve in which those who are satisfied report very low controlled eating motivation (M = 1.65), whereas females at the extremes (i.e., desire to increase, M = 2.50; moderate/severe desire to reduce, M = 2.39) experience a surge in controlled dietary regulation. The full summary of the ANOVA results is presented in Table 3.

**Figure 2:**
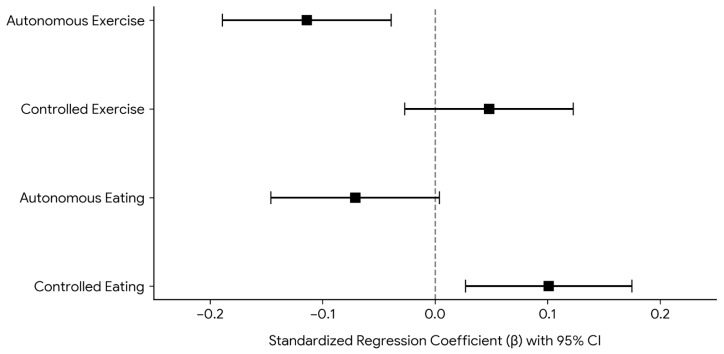
Forest-style plot displaying standardized regression coefficients and 95% Confidence Intervals for the primary predictive effect of perceived body image discrepancy on motivational outcomes. Error bars that do not cross the null line (vertical dashed line at zero) indicate statistical significance.

Furthermore, hierarchical multiple regression analyses were conducted to verify whether the primary effects of body image discrepancy remained robust after adjusting for key covariates (age, sex, and BMI). The adjusted models confirmed that perceived body image discrepancy remained a significant negative predictor of autonomous exercise motivation (β = −0.11, *p* = 0.003) and a significant positive predictor of controlled eating motivation (β = 0.10, *p* = 0.008). However, when accounting for the variance explained by the covariates (particularly BMI), the direct predictive value of body image discrepancy on controlled exercise motivation (*p* = 0.208) and autonomous eating motivation (*p* = 0.063) fell below traditional significance thresholds. This suggests that these specific associations are partially intertwined with the exercisers’ actual body mass. A visual summary of these specific direct effects, depicting the standardized regression coefficients and their respective confidence intervals, is presented in Figure 2 (for detailed step-by-step models, see Appendix A Table A1).

## 4. Discussion

The present study aimed to investigate how distinct profiles of perceived body image discrepancy relate to autonomous and controlled motivational regulations in both the exercise and eating domains, while exploring potential interaction effects with sex among regular gym exercisers. By transitionally expanding the analytical focus from a traditional dichotomous approach to a four-profile framework (i.e., desire to increase, satisfied, mild desire to reduce, and moderate/severe desire to reduce), this research offers a more nuanced understanding of the perceptual–evaluative gap as a psychological antecedent of behavioral quality. The current study expanded the analytical focus from an oversimplified binary comparison to a comprehensive four-profile framework, permitting a better mapping of how the perceptual–evaluative gap interacts with human behavioral regulation. By investigating these cross-domain pathways within a consistent sample of regular gym-goers, the findings provide insights into the correlates of exercise and eating motivations.

In line with our first hypothesis (H1), which focused on autonomous motivation for exercise, the results confirmed a significant main effect of the body image discrepancy profile. Pairwise comparisons revealed that the satisfied group reported the highest level of autonomous exercise regulation, which was significantly distinct from the Moderate/Severe Desire to Reduce group. This structural pattern aligns with the core theoretical framework of SDT [17,20] and past systematic observations [21,26]. Individuals who perceive no subjective gap between their current and ideal body figures may possess a more stable psychological foundation, viewing exercise through a lens of inherent value, identity alignment, or genuine vitality rather than as a corrective mechanism [21]. However, contrary to the expectations of H1, the main effect of sex and the interaction between sex and body image profile did not reach statistical significance. This uniform distribution suggests that across both males and females, autonomous exercise motivation is coupled with a harmonious, non-discrepant body evaluation [26], although interpretation must be treated with caution given that the observed effect size was small, indicating that body satisfaction operates alongside other broader environmental and dispositional factors within gym settings [31].

Our second hypothesis (H2), which addressed controlled motivation for exercise, was supported by the observed main effects of the body image profile. Exercisers located at both extremes of the discrepancy spectrum, those reporting a desire to increase body size and those presenting a moderate/severe desire to reduce I, exhibited significantly elevated levels of controlled regulation compared to their satisfied peers. This closely reflects the dark side of behavioral regulation conceptualized in the SDT literature [25]. A pronounced perceptual–evaluative gap appears to operate as an internal controller, generating psychological distress, body-related shame, and ego-involvement [13,15]. Within an inherently evaluative fitness environment [28,29], individuals experiencing a larger discrepancy are likely to regulate their physical training through introjected or external pressures, such as alleviating social appearance anxiety or compliance with sociocultural pressures [18,27]. The absence of a sex-based interaction here suggests that the psychological burden of a figural discrepancy affects the controlled exercise drive of men and women in a structurally parallel manner, pushing both toward maladaptive behavioral patterns [15,31].

Regarding the nutritional domain, our third hypothesis (H3) postulated that the satisfied group would exhibit the highest autonomous eating motivation and that a main effect of sex would emerge. The univariate results confirmed this pathway, revealing significant main effects for both body image profile and sex. Exercisers with a congruent body perception demonstrated a value-based, flexible approach to nutrition, supporting the notion of a positive cross-domain motivational spill-over effect between body appreciation and self-determined lifestyle practices [32,33]. Furthermore, females presented significantly higher overall levels of autonomous eating regulation than males. This distinct gap is well-supported by the gender-socialization literature, which indicates that women frequently internalize a more health-conscious, complex, and nutritionally sophisticated rationale for dietary habits, whereas men’s autonomous dietary choices are often less systematically tied to acute cognitive evaluations of wellness in recreational fitness cohorts [34,35].

The most theoretically interesting finding of this study pertains to our fourth hypothesis (H4), which anticipated a significant interaction effect between body image profiles and sex on controlled eating motivation. The statistical models revealed a highly significant interaction. While male exercisers demonstrated moderate levels of controlled eating motivation that remained stable regardless of their figural satisfaction, ranging strictly between 2.06 and 2.30, female exercisers exhibited a dramatic “U-shaped” curve. Specifically, women who were satisfied reported exceptionally low controlled eating motivation (M = 1.65), whereas those at both poles of dissatisfaction, the desire to increase (M = 2.50) and the moderate/severe desire to reduce (M = 2.39)—experienced a severe, sharp increase in rigid dietary regulation. This interaction highlights how sex-specific sociocultural pressures intersect with body image perceptions to shape behavioral vulnerability [35,36]. In contemporary Western media and fitness cultures, women are subjected to multi-layered, often contradictory body ideals, demanding both extreme thinness and functional, tonal muscularity [10,12]. When a female exerciser experiences a subjective discrepancy in either direction, this evaluative gap translates directly into guilt-driven, rigid, and introjected dietary controls to achieve the internalized ideal [22,34]. For men, although body dissatisfaction and the drive for muscularity are highly prevalent [10], these perceptions appear less systematically linked to immediate dietary guilt or rigid, controlled eating regulation in non-clinical fitness populations, maintaining a more uniform baseline threshold [36]. This divergence underscores that the impact of a perceptual gap on controlled eating is uniquely amplified by female gender socialization, validating the necessity for nuanced, sex-sensitive counseling in public health and fitness industries.

### Study Strengths, Limitations and Future Directions

This study possesses several theoretical and methodological strengths that expand the current understanding of behavioral regulation in health environments. First, it addresses an under-explored pathway by treating perceived body image discrepancy strictly as a psychological antecedent cross-domain factor rather than a mere outcome of physical practice [16]. By doing so, it simultaneously interfaces structural motivational regulations across two highly interconnected health domains, exercise and eating, within a single, ecologically valid environment [18,32]. Second, moving beyond an oversimplified binary comparison, the operationalization of a multi-profile framework captures both the direction and magnitude of the perceptual–evaluative gap [44,45]. Third, the inclusion of a large, consistent sample (N = 939) of regular gym exercisers, paired with rigorous data screening protocols (e.g., Mahalanobis distance filtering and Harman’s single-factor diagnostic clearing), ensures strong internal reliability, minimizing the risk of common method variance contaminating the observed interaction patterns.

Despite its strengths, several limitations must be addressed to contextualize the generalizability of the findings. First, the cross-sectional design precludes any causal or deterministic inferences. Although the theoretical model positions body image discrepancy as an antecedent to autonomous and controlled regulations, bidirectional or reciprocal pathways are highly plausible [31,32]. Longitudinal and experimental methodologies are strictly required to map true causal trajectories over time. Furthermore, because cross-domain data regarding both exercise and eating regulations were collected from the same respondents at a single time-point using analogous Likert-scale formats, shared method variance might inflate the observed associations, an issue that baseline diagnostics such as Harman’s test cannot entirely eliminate. Second, the final sample comprised exclusively active gym members from a specific geographic region who had passed the six-month exercise maintenance threshold [8]. Consequently, these motivational dynamics may not extend to sedentary populations, individuals initiating fitness programs, or clinical cohorts with severe eating or body dysmorphic disorders. Third, a specific analytical trade-off must be acknowledged regarding construct validity. Body image evaluation was measured exclusively via the perceptual–evaluative discrepancy score derived from the FRS [42]. While the FRS is a pragmatic, validated, and predictive tool for large-scale studies [43], it focuses on a single dimension of body size evaluation and fails to capture the broader multidimensionality (i.e., affective, cognitive, and behavioral dimensions) of the human body image experience [10,11]. Fourth, from a statistical perspective, the initial five-level classification of discrepancy was reduced to four final analytical profiles by collapsing the two lowest scores reflecting a desire to increase body size. This operational decision was enforced due to the highly unbalanced nature of the raw cell distributions, a common trend in general fitness environments where thin-ideal and reduction goals predominate, to avoid severe violations of statistical power and homoscedastic cell assumptions in the factorial models. Although mathematically justified to preserve inferential validity, this fusion limits the assessment of whether moderate versus severe desire to increase body size operates under distinct motivational thresholds. Future investigations should oversample individuals desiring larger body sizes to validate these sub-dimensions independently. Finally, the uniformly small effect sizes observed across the univariate models clearly indicate that perceived body image discrepancy represents a singular piece within a vast, multi-determined motivational ecosystem [31]. Future researchers should avoid narrow, isolated approaches and instead integrate other complementary psychological variables, such as self-compassion, ego-oriented perfectionism, and explicit weight-stigma scales, that likely interact with the perceptual–evaluative gap to shape exercise persistence and long-term nutritional health.

## 5. Conclusions

This study demonstrates that perceived body image discrepancy profiles are significant, though modestly, associated with the quality of motivation in both the exercise and eating domains among regular gym exercisers. Grounded in SDT, our findings indicate that body satisfaction, the complete absence of a perceptual gap, represents a relevant psychological correlate of highly adaptive, autonomous behavioral regulation across health domains. Conversely, experiencing a discrepancy in either direction, wishing to be larger or smaller, is consistently linked to elevated controlled motivation. The relationship between body image discrepancy and controlled eating motivation is moderated by biological sex. While males maintain stable controlled eating motivation across all profiles, females exhibit a distinct “U-shaped” vulnerability, experiencing a severe increase in rigid, controlled dietary regulation at both extremes of dissatisfaction. Methodologically, the small-to-moderate magnitude of all observed effect sizes requires a non-deterministic interpretation, highlighting that body image discrepancy is a single factor within a vastly complex and multi-determined motivational ecosystem. Practically, these findings imply that public health strategies and fitness professionals should actively pivot away from appearance-focused or weight-centric discourse.

Instead, practitioners must implement concretely differentiated, sex-tailored strategies to foster need-supportive environments. For female exercisers, who exhibit heightened vulnerability to rigid dieting at both extremes of body dissatisfaction, professionals should provide feedback that actively decouples nutritional choices from appearance-based guilt, focusing instead on food as nourishment for energy, recovery, and genuine vitality. Conversely, for male exercisers, who display more uniform but moderate controlled eating drives regardless of body size, feedback should aim to shift their focus from broad, sociocultural functional ideals toward building inherent competence and autonomous value in long-term nutritional health.

## Figures and Tables

**Table 1:** Sociodemographic and training characteristics of the participants (N = 939).

Variables	Category	n (%) or M ± SD
Sex	Female	517 (55.1%)
	Male	422 (44.9%)
Age (years)		32.99 ± 11.90
Educational Attainment	Basic Education	32 (3.4%)
	High School	321 (34.2%)
	Bachelor’s Degree	435 (46.3%)
	Master’s Degree	134 (14.3%)
	PhD	17 (1.8%)
Exercise Experience (months)		30.72 ± 45.75
Weekly Training Frequency		3.76 ± 1.28
Primary Exercise Modality	Resistance Training	501 (53.4%)
	Group Classes	286 (30.5%)
	Personal Training	114 (12.1%)
	Aquatic Activities	6 (0.6%)
	Other	32 (3.4%)

Note. M = Mean; SD = Standard Deviation. The “Resistance Training/Gym Floor” category includes independent workouts. Educational categories were adjusted to reflect standard international classifications.

**Table 2:** Descriptive statistics of exercise and eating motivation by body image discrepancy profile and sex.

Variables	Autonomous Motivation (Exercise)	Controlled Motivation (Exercise)	Autonomous Motivation (Eating)	Controlled Motivation (Eating)
Body Image Discrepancy Profile	M ± SD	M ± SD	M ± SD	M ± SD
Desire to increase (n = 131)	3.42 ± 0.51	1.07 ± 0.55	5.60 ± 0.84	2.31 ± 1.03
Satisfied (n = 272)	3.46 ± 0.50	0.83 ± 0.56	5.75 ± 1.04	1.85 ± 0.86
Mild desire to reduce (n = 324)	3.33 ± 0.51	0.92 ± 0.54	5.62 ± 1.00	2.09 ± 0.90
Moderate/Severe desire to reduce (n = 212)	3.23 ± 0.57	1.08 ± 0.52	5.38 ± 1.04	2.35 ± 0.90
Sex				
Female (n = 517)	3.34 ± 0.52	0.93 ± 0.55	5.71 ± 0.98	2.05 ± 0.94
Male (n = 422)	3.38 ± 0.54	0.98 ± 0.55	5.46 ± 1.02	2.18 ± 0.90
Total Sample (N = 939)	3.36 ± 0.53	0.95 ± 0.55	5.60 ± 1.01	2.11 ± 0.93

Note. M = Mean; SD = Standard Deviation. Autonomous and controlled motivation for exercise were measured on a 0–4 scale. Autonomous and controlled motivation for eating were measured on a 1–7 scale. Higher scores indicate higher levels of the respective motivational regulation.

**Table 3:** MANOVA results for the effects of body image profile and sex on exercise and eating Motivation.

Dependent Variable	Source	df	F	*p*	η^2^_p_
Autonomous Mot. (Exercise)	Body Image (Group)	3	8.97	<0.001 ***	0.028
	Sex	1	1.32	0.251	0.001
	Interaction (Group × Sex)	3	2.26	0.080	0.007
Controlled Mot. (Exercise)	Body Image (Group)	3	11.02	<0.001 ***	0.034
	Sex	1	0.35	0.553	0.000
	Interaction (Group × Sex)	3	1.31	0.269	0.004
Autonomous Mot. (Eating)	Body Image (Group)	3	5.89	<0.001 ***	0.019
	Sex	1	11.74	<0.001 ***	0.012
	Interaction (Group × Sex)	3	0.88	0.450	0.003
	Body Image (Group)	3	14.90	<0.001 ***	0.046
	Sex	1	0.78	0.376	0.001
	Interaction (Group × Sex)	3	6.22	<0.001 ***	0.020

Note. df = degrees of freedom; η^2^_p_ = partial eta-squared. Post hoc comparisons reflect the main effect of body image profile across the four groups. (***) denotes *p* < 0.001. Mod/Severe = moderate to severe.

## Data Availability

The data presented in this study are available on request from the corresponding author. The data are not publicly available due to privacy and ethical restrictions regarding participant anonymity.

## Appendix A

**Table A1:** Hierarchical multiple regression models predicting exercise and eating motivation, adjusted for age, sex, and BMI.

Predictors	Autonomous Exercise β [95% CI]	Controlled Exercise β [95% CI]	Autonomous Eating β [95% CI]	Controlled Eating β [95% CI]
Step 1 (Covariates)				
Age	0.09 [0.001, 0.007] **	−0.12 [−0.009, −0.003] ***	0.12 [0.005, 0.016] ***	−0.20 [−0.021, −0.011] ***
Sex	0.04 [−0.029, 0.114]	0.02 [−0.047, 0.101]	−0.11 [−0.352, −0.082] **	0.05 [−0.020, 0.226]
BMI	−0.06 [−0.021, 0.002]	0.10 [0.003, 0.027] *	−0.07 [−0.043, 0.001]	0.06 [−0.004, 0.036]
Step 2 (Primary Predictor)				
Body Image Discrepancy	−0.11 [−0.081, −0.016] **	0.05 [−0.012, 0.055]	−0.07 [−0.118, 0.003]	0.10 [0.020, 0.131] **
Model Fit				
Total R^2^	0.03	0.03	0.04	0.05
Delta R^2^ (Step 2)	0.01	0.00	0.00	0.01
F	F(4, 934) = 6.62 ***	F(4, 934) = 6.43 ***	F(4, 934) = 9.67 ***	F(4, 934) = 13.38 ***

Note. β = standardized regression coefficient from the final model (Step 2); CI = unstandardized confidence interval; BMI = Body Mass Index. Body Image Discrepancy was entered as a continuous score (Current—Ideal). To optimize presentation, covariates’ coefficients reflect the final adjusted model. * *p* < 0.05, ** *p* < 0.01, *** *p* < 0.001.

## References

[1] Warburton D.E.R., Bredin S.S.D. (2017). Health benefits of physical activity: A systematic review of current systematic reviews. Curr. Opin. Cardiol..

[2] Guthold R., Stevens G.A., Riley L.M., Bull F.C. (2018). Worldwide trends in insufficient physical activity from 2001 to 2016: A pooled analysis of 358 population-based surveys with 1.9 million participants. Lancet Glob. Health.

[3] Guthold R., Stevens G.A., Riley L.M., Bull F.C. (2020). Global trends in insufficient physical activity among adolescents: A pooled analysis of 298 population-based surveys with 1.6 million participants. Lancet Child Adolesc. Health.

[4] World Health Organization Nearly 1.8 Billion Adults at Risk of Disease from Not Doing Enough Physical Activity. https://www.who.int/news/item/26-06-2024-nearly-1.8-billion-adults-at-risk-of-disease-from-not-doing-enough-physical-activity.

[5] Bull F.C., Al-Ansari S.S., Biddle S., Borodulin K., Buman M.P., Cardon G., Carty C., Chaput J.P., Chastin S., Chou R. (2020). World Health Organization 2020 guidelines on physical activity and sedentary behaviour. Br. J. Sports Med..

[6] European Commission Eurobarómetro Portugal—Desporto e Atividade Física 2022. https://europa.eu/eurobarometer/surveys/detail/2668.

[7] Gust A. (2024). Effect of health conditions and community program participation on physical activity and exercise motivation in older adults. J. Health Psychol..

[8] Rodrigues F., Teixeira D.S., Cid L., Monteiro D. (2021). Have you been exercising lately? Testing the role of past behavior on exercise adherence. J. Health Psychol..

[9] Sabiston C.M., Pila E., Vani M., Thogersen-Ntoumani C. (2019). Body image, physical activity, and sport: A scoping review. Psychol. Sport Exerc..

[10] Grogan S. (2021). Body Image: Understanding Body Dissatisfaction in Men, Women and Children.

[11] Cash T.F. (2011). Body Image: A Handbook of Science, Practice, and Prevention.

[12] Merino M., Tornero-Aguilera J.F., Rubio-Zarapuz A., Villanueva-Tobaldo C.V., Martín-Rodríguez A., Clemente-Suárez V.J. (2024). Body perceptions and psychological well-being: A review of the impact of social media and physical measurements on self-esteem and mental health with a focus on body image satisfaction and its relationship with cultural and gender factors. Healthcare.

[13] Rudiger J.A., Cash T.F., Roehrig M., Thompson J.K. (2007). Day-to-day body-image states: Prospective predictors of intra-individual level and variability. Body Image.

[14] Üstündağ A., Kurtkaya K., Zaman Ö. (2026). The relationship between adolescents’ body image, social appearance anxiety, and motivation to participate in physical activity: A cross-sectional study. J. Health Psychol..

[15] Kalanava T.V., Maes S., Gucht V. (2015). Interpersonal and Self-regulation Determinants of Healthy and Unhealthy Eating Behavior in Adolescents. J. Health Psychol..

[16] Campbell A., Hausenblas H.A. (2009). Effects of Exercise Interventions on Body Image: A Meta-analysis. J. Health Psychol..

[17] Deci E.L., Ryan R.M. (2000). The “What” and “Why” of Goal Pursuits: Human Needs and the Self-Determination of Behavior. Psychol. Inq..

[18] Salvador R., Monteiro D., Rebelo-Gonçalves R., Jiménez-Castuera R. (2023). Interpersonal behavior, basic psychological needs, motivation, eating behavior, and body image in gym/fitness exercisers: A systematic review. Sustainability.

[19] Ajzen I. (2011). The theory of planned behaviour: Reactions and reflections. Psychol. Health.

[20] Ryan R.M., Deci E.L. (2017). Self-Determination Theory: Basic Psychological Needs in Motivation, Development, and Wellness.

[21] Teixeira P.J., Carraça E.V., Markland D., Silva M.N., Ryan R.M. (2012). Exercise, physical activity, and self-determination theory: A systematic review. Int. J. Behav. Nutr. Phys. Act..

[22] Ntoumanis N., Ng J.Y.Y., Prestwich A., Quested E., Hancox J.E., Thøgersen-Ntoumani C., Deci E.L., Ryan R.M., Lonsdale C., Williams G.C. (2021). A meta-analysis of self-determination theory-informed intervention studies in the health domain: Effects on motivation, health behavior, physical, and psychological health. Health Psychol. Rev..

[23] Edmunds J., Ntoumanis N., Duda J.L. (2006). A test of self-determination theory in the exercise domain. J. Appl. Soc. Psychol..

[24] Rhodes R.E., Kates A. (2015). Can the Affective Response to Exercise Predict Future Motives and Physical Activity Behavior? A Systematic Review of Published Evidence. Ann. Behav. Med..

[25] Rodrigues F., Teixeira D.S., Cid L., Machado S., Monteiro D. (2019). The role of dark-side of motivation and intention to continue in exercise: A self-determination theory approach. Scand. J. Psychol..

[26] Panão I., Carraça E.V. (2020). Effects of exercise motivations on body image and eating habits/behaviours: A systematic review. Nutr. Diet..

[27] Vartanian L.R., Dey S. (2013). Self-concept clarity, thin-ideal internalization, and appearance-related social comparison as predictors of body dissatisfaction. Body Image.

[28] Rocchi M., Pelletier L., Cheung S., Baxter D., Beaudry S. (2017). Assessing need-supportive and need-thwarting interpersonal behaviours: The Interpersonal Behaviours Questionnaire (IBQ). Personal. Individ. Differ..

[29] Rodrigues F., Pelletier L.G., Rocchi M., Neiva H.P., Teixeira D.S., Cid L., Silva L., Monteiro D. (2021). Trainer-exerciser relationship: The congruency effect on exerciser psychological needs using response surface analysis. Scand. J. Med. Sci. Sports.

[30] Rodrigues F., Monteiro D., Teixeira D., Cid L. (2020). Exercise professionals role on exercise adherence in Portugal: The importance of need-supportive behaviors and motivational climates. Motricidade.

[31] Brunet J., Gunnell K.E., Gaudreau P., Sabiston C.M. (2015). An integrative analytical framework for understanding the effects of autonomous and controlled motivation. Personal. Individ. Differ..

[32] Guertin C., Pelletier L.G., Émond C., Lalande G. (2017). Change in physical and psychological health over time in patients with cardiovascular disease: On the benefits of being self-determined, physically active, and eating well. Motiv. Emot..

[33] Pelletier L.G., Dion S.C., Slovinec-D’Angelo M., Reid R. (2004). Why do you regulate what you eat?. J. Appl. Soc. Psychol..

[34] Verstuyf J., Vansteenkiste M., Soenens B. (2012). Eating regulation and bulimic symptoms: The differential correlates of health-focused and appearance-focused eating regulation. Body Image.

[35] Nimiya A., Vasudha K.G., Shetty S.B., Pai K., Reshma N.S., Radhika K., D’Souza M., D’Souza P. (2024). Sex difference in body image, exercise motivation and social comparison among Instagram users: A cross sectional study. F1000Research.

[36] Sheng J., Ariffin I.A.B., Tham J. (2025). The influence of exercise self-efficacy and gender on the relationship between exercise motivation and physical activity in college students. Sci. Rep..

[37] Faul F., Erdfelder E., Lang A.G., Buchner A. (2007). G*Power 3: A flexible statistical power analysis program for the social, behavioral, and biomedical sciences. Behav. Res. Methods.

[38] Cid L., Monteiro D., Teixeira D.S., Teques P., Alves S., Moutão J., Silva M., Palmeira A. (2018). The Behavioral Regulation in Exercise Questionnaire (BREQ-3) Portuguese-version: Evidence of reliability, validity and invariance across gender. Front. Psychol..

[39] Markland D., Tobin V. (2004). A modification to the behavioural regulation in exercise questionnaire to include an assessment of amotivation. J. Sport Exerc. Psychol..

[40] Wilson P.M., Rodgers W.M., Loitz C.C., Scime G. (2006). “It’s who I am... really!”: The importance of integrated regulation in exercise contexts. J. Appl. Biobehav. Res..

[41] Teixeira D.S., Pelletier L., Encantado J., Marques M.M., Rodrigues B., Carraça E.V. (2021). Adaptation and validation of the Portuguese version of the regulation of eating behavior scale (REBSp). Appetite.

[42] Stunkard A.J., Sorensen T., Schulsinger F., Kety S. (1983). Use of the Danish Adoption Register for the study of obesity and thinness. The Genetics of Neurological and Psychiatric Disorders.

[43] Scagliusi F.B., Alvarenga M., Polacow V.O., Cordás T.A., de Oliveira Queiroz G.K., Coelho D., Philippi S.T., Lancha A.H. (2006). Concurrent and discriminant validity of the Stunkard’s figure rating scale adapted into Portuguese. Appetite.

[44] Gardner R.M., Brown D.L. (2010). Body image assessment: A review of figural drawing scales. Personal. Individ. Differ..

[45] Thompson M.A., Altabe M.N. (1991). Psychometric qualities of the Figure Rating Scale. Int. J. Eat. Disord..

[46] Cohen J. (1983). The cost of dichotomization. Appl. Psychol. Meas..

[47] Thompson M.A., Gray J.J. (1995). Development and validation of a new body-image assessment scale. J. Personal. Assess..

[48] Cohen J., Hillsdale N.J. (1988). Statistical Power Analysis for the Behavioral Sciences.

[49] Ho R. (2014). Handbook of Univariate and Multivariate Data Analysis and Interpretation with SPSS.

